# Your Eyes Do Not Lie! Dissecting Humor Effects in Health Messages Using Eye Tracker Technology

**DOI:** 10.3389/fpubh.2021.653584

**Published:** 2021-05-25

**Authors:** Emmanuelle Brigaud, Alex Lafont, Nathalie Blanc

**Affiliations:** ^1^Université Paul Valéry Montpellier 3, Laboratory Epsylon EA 4556, Montpellier, France; ^2^Institut Supérieur de l'Aéronautique et de l'Espace, Toulouse, France

**Keywords:** humor, health communication, visual attention, eye-tracking technology, preventive messages

## Abstract

In the past decade, humor in scientific research has become more and more popular providing an increase of data identifying the context in which humor is a promising communication strategy in preventive health messages. To avoid the limits of declarative responses usually recorded in past studies, eye tracker technology offers the possibility to assess and dissect the effects of humor on visual attention. In this brief report, we first attempt to extend the results of previous studies by recording eye movements while participants were exposed to humorous and nonhumorous print health ads dealing with tobacco and alcohol consumption. A secondary purpose is specifically to test the visual attention French women devoted to humorous tobacco preventive ads, the worrying results of recent studies urging to find a way to improve tobacco preventive campaigns. Based on three complementary eye-tracking measures (i.e., total dwell time, fixation count, and revisits), the results showed that humorous health messages were scanned longer and more frequently and revisited more often compared to nonhumorous ones. In addition, humor appeared to reduce smokers' avoidance of preventive tobacco messages. The different pattern of visual exploration confirms that humor is a good strategy to grab attention even of individuals who are involved in the health topic addressed. In short, this paper argues for introducing lightness into a very serious subject, health communication, based on the analysis of eye movement evidence.

## Introduction

The positive effects of humor have been widely demonstrated in the advertising context [see (1), for a review) and, more recently, in ads for public health issues ranging from consumption of tobacco and alcohol, to cancer prevention and screening, to sexually transmitted diseases [e.g., (2–6)]. Humor may be one effective way to gain audience attention toward health messages and to enhance the processing of central information in preventive health campaigns. Previous studies by Blanc and Brigaud (7) compared humorous and nonhumorous preventive health message in print ads dealing with three topics (tobacco, alcohol, and obesity). They showed that the presence of humor attracted attention and promoted the memory storage of health messages.

There are theoretical reasons to assume that humor receives more attention and more elaborate processing than nonhumor in preventive health campaigns. First, in this context, humorous stimuli are unexpected because health ads rely on unhealthy and risky behavior. Because the ad content differs from the normality or the audience expectation, humor arouses surprise and presents a challenge to be resolved. For example, consider a preventive health ad dealing with tobacco previously used in experimental studies: “The picture shows a cemetery with a green lawn and many white crosses. At the center of the picture, there is an empty space where there are no crosses but only a health message that indicates non-smokers area” (7). This nonconventional message attracts attention and requires audience effort to resolve its incongruity, a necessary step to access its subsequent humorous evaluation [i.e., (8–10)]. The incongruity resolution involved in humor processing demands an additional processing effort compared to nonhumorous stimuli. Consequently, to access the humorous meaning, participants take longer to explore ads that make them laugh. According to this explanation, (7) showed evidence for the fact that humor inserted in health ads attracted individuals' attention and involved a more efficient storage of preventive messages suggesting that this extra viewing time reflected a deeper processing of humorous messages.

Secondly, humor reduces negative emotional reactions to unappealing topics (11, 12) and decreases people's defensive mechanism that may interfere with the processing of health messages. In other words, affective responses to humor (e.g., happiness, fun, pleasure) lead to decreased reactance and increased persuasiveness of the health messages [e.g., (13)]. For example, (14) showed that when exposed to an entertainment narrative on unplanned pregnancy, female viewers who saw a humorous version (as opposed to a nonhumorous one) reported increased severity of the depicted negative consequences of risky behaviors. Moreover, positive affect evoked by the presence of humor leads to reduced motivation to engage in a critical disagreement of the message arguments, making the persuasive message more efficient [e.g., (15, 16)]. One recent study conducted by (17) revealed that humor has the power to reduce message resistance even among audiences who disagree with the underlying content. In this study, vaccine-hesitant parents exposed to a message presenting the importance of the vaccination with a satirical message reported less reactance and less vaccine hesitancy compared to those exposed to a more serious message. In summary, the presence of humor in health messages attracts attention and makes the audience more open and less critical to health recommendations.

If humor seems to promote health message acceptance, the fact that humor can have the power to attract attention relies mostly on indirect evidence. Empirical evidence supporting the impact of humor on attention has focused widely on offline (i.e., afterward) memory performance. For example, in Blanc and Brigaud's (7) studies, individuals spent longer time viewing humorous health ads compared to nonhumorous ones and, consequently, better recalled humorous messages. This pattern of results should be taken only as indirect evidence of the stopping power of humor since it is unclear whether individuals actually fixated on the humorous health messages more than on the nonhumorous ones and whether extra humorous ads viewing time always means that humorous messages require higher processing demands. The use of an objective method like eye-tracking enables to directly capture visual attention devoted to health messages and thus contributes to deepen our understanding of how the presence of humor benefits to health messages.

Nowadays, eye-tracking systems are valuable tools used to study the factors affecting the ability of ads to gain attention and to evaluate communication effectiveness [see (18), for a review]. In health communication, a large number of studies have investigated the importance of eye gaze to improve the understanding of message effectiveness in various health topics: tobacco [see (19) for a review], cancer [e.g., (20, 21), and binge drinking (22). However, to our knowledge, none of these studies has considered humor effect in health communication using eye-tracking technology. At least there are promising results already reported in the commercial advertising with the study conducted by (23). Their results showed that in commercial ads humorous messages received more attention than nonhumorous ones. Note that in this study, analysis of eye movements was based only on the total amount of time spent looking at the area in which messages were presented. So, this total dwell time was the only indication of individuals' attention pattern viewing humorous information. Other eye-tracking metrics are now very valuable to allow researchers to provide information about how humorous content is prioritized and processed [e.g., (24–26)].

Precisely, there are at least three complementary measures that eye-tracking researchers analyze. The number of times (fixations count) and how long (dwell time) a respondent looks at a specific area of an ad can provide direct evidence to the stopping power of this area and can also uncover the amount of processing being applied to this area [e.g., (27)]. Likewise, the number of revisits to a specific area (i.e., the number of times that a respondent returns to an area of interest) can indicate that this area has a better attention-getting property. It has to be underlined that the link between eye-tracking measures of attention and advertising effectiveness had already been evidenced not only in commercial advertising but also in health communication. Indeed, (18) reported fruitful links in commercial ads between visual attention and consumer cognition (comprehension, memory), affect (attitudes, liking), and behavior (choice, purchase). Similarly (19), revealed that greater visual attention (i.e., more fixations and longer dwell time) to health messages was associated with higher cognitive processing and better recall of the warnings. Note that viewing patterns are also associated with perceptions of message convincingness and pleasantness (22).

Based on the idea that eye-tracking measures offer an objective insight to complement results suggesting that humor increases attention, recall, and effectiveness of health messages, the present study proposes to record eye movements while participants are exposed to humorous and nonhumorous preventive messages. Because humorous health messages are nonconventional messages and require audience effort to resolve their inherent incongruity, we assume that longer dwell time and higher number of fixations should be observed in favor of humorous messages compared to nonhumorous ones. Also, we hypothesize that if health messages with a humorous content promote audience interest and elicit positive emotions, then they should grab attention and therefore should be revisited more often than nonhumorous ones.

To reinforce the idea that humor is a useful strategy in health communication and to overcome the limits of indirect evidence already provided by previous works, the present study attempts to extent Blanc and Brigaud's (7) contribution on humour effect on individuals' attention using eye-tracking technology. In addition, the present study pursues a secondary objective that takes into account worrying current trends in France related to tobacco consumption in women. According to (28), the incidence of lung cancer increased by 72% among women between 2002 and 2015. This large increase urges French health policies to prevent tobacco consumption in young females. The present study directly addresses the necessity to improve tobacco prevention in French women, considering this target audience to capture humor effects in preventive messages using eye-tracker technology. According to (19), consumers spend little time attending to health warnings on tobacco ads or completely ignore or avoid warnings. In the present study, recording eye movements offers the advantage to directly test whether humor can counteract this visual avoidance of preventive messages on the target audience (i.e., smokers).

## Materials and Methods

### Participants

Sixty French female undergraduate students took part in the experiment (Age_M_ = 19.22, SD = 1.67). All participants had normal or corrected-to-normal vision and gave formal consent to participate in this study. The participants were volunteers who received extra credit in psychology classes for their participation. In our sample, we distinguished smokers (*n* = 24) from nonsmokers (*n* = 36) based on a short questionnaire administrated at the end of the experiment.

### Materials

To compare the attention allocation in time and space devoted to humorous and nonhumorous health messages, we selected 12 print health ads dealing with alcohol (*n* = 6) and tobacco (*n* = 6) from Blanc and Brigaud's (7) studies. Using this material presented two advantages: first, its validity had already been checked, and second, it enabled us to extend on previous results. For each health ad (whatever the topic), a humorous version and a nonhumorous version were compared. Precisely, the two versions of each ad differed only in the type of preventive message (humorous vs. nonhumorous) that was inserted. As for example, remind the tobacco preventive ad described in the introduction: It was composed of a visual exhibiting a cemetery with a green lawn and many white crosses all over. At the center of this visual, there was an empty space where there were no crosses. The health message written in this space was either humorous (i.e., Non-smokers area) or nonhumorous (i.e., Tobacco kills you). The way these messages were displayed on the visual was identical (e.g., font, size, location) in order to examine possible differences in attention given to each of them. To assess the smoking status of the participants, we used a two-item questionnaire. The first question focused on the cigarette consumption (i.e., how many cigarettes/day do you smoke?), and the second question targeted the severity of the dependence to tobacco (i.e., how soon after you wake up do you smoke your first cigarette?). According to (29), participants declaring to smoke at least five cigarettes a day and within 1 h of waking were considered as smokers.

### Apparatus

Stimuli were presented on a 19-inch screen with a 1,440 × 900 pixel resolution, using the software Experiment Center 3.5 from SensoMotoric Instruments (SMI, Teltow, Germany). Participants' eye movements were recorded using a video-based remote eye-tracking system (SMI iView X™ RED-m; 120 Hz sampling rate) and the corresponding SMI software iView X™. The system was calibrated by IView following a nine-point calibration. Note that the SMI RED-m system is a tool with full flexibility and mobility and its higher sampling frequency (120 Hz) ensures accurate and reliable results from event detection.

### Procedure

Participants were received individually and started by completing an informed consent. Then, participants were placed in a comfortable sitting position in front of the RED module and the stimulus monitor at a distance of ~70 centimeters (the recommended distance). They were asked to limit head movements during the eye-tracking session. Before the experiment started, the calibration quality was assessed automatically with a self-programmed nine-point validation. Following this, participants were invited to watch as they normally would a series of preventive health ads on the computer screen. Before the presentation of each ad, participants saw a blank fixation screen lasting 500 ms. Each ad was automatically displayed on the computer screen for 8 s, corresponding to the amount of time individuals spent watching each of them [see (7)]. All participants were exposed to the two topics (tobacco and alcohol). To ensure that participants saw only one version of each ad (humorous vs. nonhumorous one) and were exposed to the same number of humorous and nonhumorous ads per health topic, the humorous and nonhumorous versions of each ad were counterbalanced across them. To avoid any effect of the topic, and like in our previous study [experiment 2, (7)], half of the participants started with tobacco topic while the other half started with alcohol topic. In sum, a total set of 12 ads was explored by each participant, namely, six tobacco ads (three humorous and three nonhumorous) and six alcohol ads (three humorous and three nonhumorous). Note that within each series, ads were shown in mixed order to all participants. At the end of the experiment, each participant had to respond to the tobacco habits questionnaire.

### Measures

The eye-movement data were analyzed with Begaze™ software, version 3.5, from SMI. A fixation was detected when eye movements stayed for at least 80 milliseconds on a position with a maximum dispersion of 100 pixels. To measure how attention was allocated across the humorous and nonhumorous preventive health ads, we defined one a-priori area of interest (AOI) that covered health messages. Fixations on other areas were ignored.

For this AOI, three common eye-tracking measures were considered: total dwell time (i.e., the total amount of time that a participant spent looking at the AOI), mean fixation count (i.e., the average number of fixations detected within the AOI), and number of revisits (i.e., the number of times the AOI was revisited after the first visit). These three eye-movement measures were automatically calculated for each participant by the Data Viewer software.

## Results

Repeated-measure analyses of variance (ANOVAs) were run on three eye movement measures with the type of advertisements (with humorous message vs. with nonhumorous message) and the topic of the ads (alcohol, tobacco) as within-participant factors.

Participants' gaze varied according to type of advertisements as shown by significant main effects on three eye-tracking measures (Table 1).

**Table 1:** Summary of means along with standard deviations of eye-tracking measures for humorous and nonhumorous preventive health ads.

		**Type of advertisements**			
		**Humorous**		**Nonhumorous**	
**Measures**	N	M	SD	M	SD
Total dwell time (*ms*)	60	3253.72	775.82	2622.19	753.24
Fixation count	60	11.15	2.53	9.25	2.89
Revisits	60	3.17	0.72	2.69	0.70

Type of advertisements affected were total dwell time *F* (1,118) = 20.47, *p* < 0.001, $\eta_{\text{p}}^{2}$ = 0.15, mean fixation count *F* (1,118) = 18.68, *p* < 0.001, $\eta_{\text{p}}^{2}$ = 0.14, and revisits *F* (1,118) = 14.04, *p* < 0.001, $\eta_{\text{p}}^{2}$ = 0.11. As expected, the total amount of time spent within the AOI, the average number of fixations detected in the AOI, and the number of revisits were significantly higher for ads with humorous message compared to ads with nonhumorous message.

A significant main effect of ad topic was also observed for total dwell time *F* (1,118) = 16.54, *p* < 0.001, $\eta_{\text{p}}^{2}$ = 0.12 and mean fixation count *F* (1,118) = 43.93, *p* < 0.001, $\eta_{\text{p}}^{2}$ = 0.27. Participants spent more time looking at the AOI for alcohol preventive health ads (*M* = 3104.65 ms, *SD* = 1000.69) compared to those dealing with tobacco (*M* = 2771.26 ms, *SD* = 875.56) and there were more eye fixations toward alcohol preventive health ads (*M* = 11.15, *SD* = 3.52) than toward those dealing with tobacco (*M* = 9.23, *SD* = 2.45). Analyses of the eye tracking measures did not reveal any other significant results.

### Supplementary Analysis

To gain additional insights on humor effects on tobacco ads depending on individuals' involvement (smokers vs. nonsmokers), we conducted three additional analyses only on this health topic comparing the humorous and nonhumorous versions.

ANOVAs showed significant differences in means due to type of advertisements for total dwell time *F* (1,116) = 11.22, *p* = 0.001, $\eta_{\text{p}}^{2}$ = 0.09; mean fixation count *F* (1,116) = 17.27, *p* < 0.001, $\eta_{\text{p}}^{2}$ = 0.13; and revisits *F* (1,116) = 17.90, *p* < 0.001, $\eta_{\text{p}}^{2}$ = 0.13. Again, the total amount of time spent within the AOI (*M*_humorous_ = 3025.72, *SD* = 908.81; *M*_nonhumorous_ = 2516.80, *SD* = 767.18), the average number of fixations detected in the AOI (*M*_humorous_ = 10.07, *SD* = 2.31; *M*_nonhumorous_ = 8.40, *SD* = 2.30), and the number of revisits (*M*_humorous_ = 3.27, *SD* = 0.89; *M*_nonhumorous_ = 2.70, *SD* = 0.83) were significantly higher for ads with a humorous message compared to ads with a nonhumorous message.

**Figure 1:**
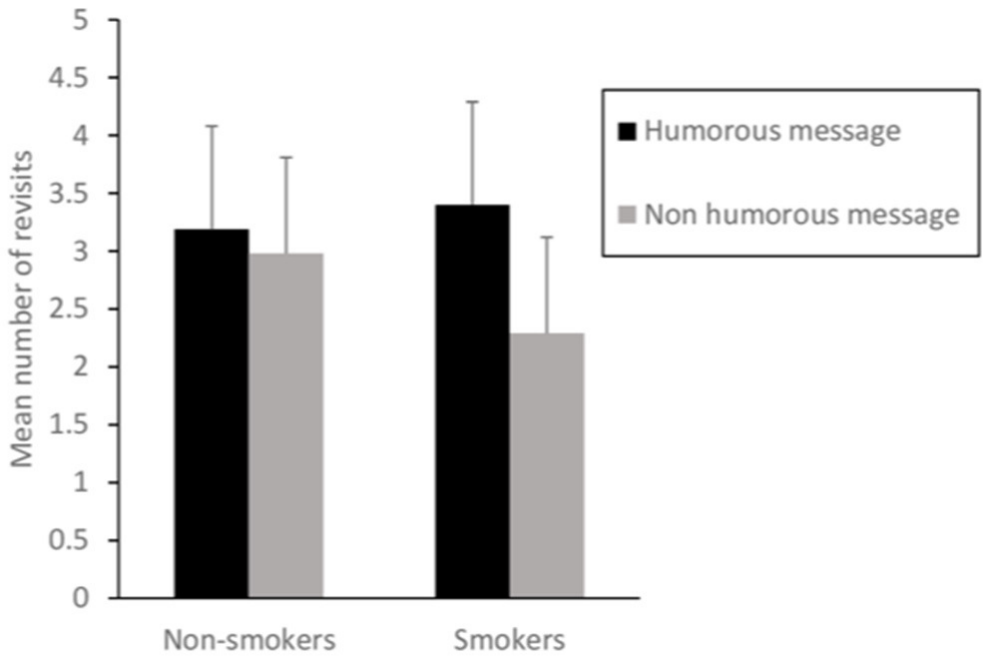
Interaction effect between type of ads and individuals' involvement. Standard errors are represented by the error bars attached to each column.

Interestingly, a significant interaction appeared between type of advertisements and individuals' involvement for revisits only, *F* (1,116) = 8.34, *p* = 0.005, $\eta_{\text{p}}^{2}$ = 0.07. As shown in Figure 1, smokers and nonsmokers differed in their number of revisits especially for nonhumorous message: the number of revisits was significantly lower for smokers (*M* = 2.29, *SD* = 0.63) compared to nonsmokers (*M* = 2.98, *SD* = 0.85), *p* < 0.05. In addition, if the two types of ads gave rise to similar patterns of revisits in nonsmokers (*M*_humorous_ = 3.19, *SD* = 0.95; *M*_nonhumorous_ = 2.98, *SD* = 0.85), *p* = 0.77, the smokers returned less to AOIs with a nonhumorous message (*M* = 2.29, *SD* = 0.63) compared to AOIs with a humorous message (*M* = 3.40, *SD* = 0.81), *p* < 0.001. Finally, smokers and nonsmokers exhibited a similar number of revisits for humorous messages (*M*_smokers_ = 3.40, *SD* = 0.81; *M*_nonsmokers_ = 3.19, *SD* = 0.95), *p* = 0.82.

## Discussion

The objective of this study was to explore the effect of humor on attention devoted to health messages by collecting eye-tracking measures. To test the idea that humor attracts individuals' attention, we recorded visual attention for humorous and nonhumorous messages. To gain further insights on previous results that reported a benefit in using humor in health communication [e.g., (2–4, 7, 17), we examined how participants visually process a series of preventive ads dealing with tobacco and alcohol comparing humorous and nonhumorous versions. Based on three complementary eye-tracking measures (i.e., total dwell time, fixation count, and revisits), we attempted to assess whether attention devoted to health messages increases in the presence of humor. Overall, our findings showed that humorous messages were scanned longer and more frequently and revisited more often compared to nonhumorous ones. This supports the power of humor to attract individuals' attention on preventive messages, a central component of health ads.

The results of the present study were notable in several ways. First, we underlined the relevance of recording eye movements to better assess how the presence of humor affects the attention allocation as it was already pointed out in the commercial area by (23). Second, we extended their results by considering not only total amount of time devoted to message but also complementary eye-tracking indicators which enable us to provide a coherent pattern of visual exploration. Third, regarding previous results dealing with humor effects in the health area (7), our data make clear that extra viewing time already observed for humorous ads compared to nonhumorous ones corresponds to increased attention allocation to the message itself. Fourth, using eye-tracking technology offers an objective picture of how individuals allocate their attention while processing print health ads. This implicit measure complements existing methods used to assess humor effects in health communication. Fifth, the benefits of using humor on tobacco ads were more salient with smokers who revisited more often humorous messages compared to nonhumorous ones. This result suggests that humor could counteract the visual avoidance of tobacco health warnings observed in smokers on cigarette packs [e.g., (19, 29–31)]. With respect to a worrying increase in lung cancer recently reported in French women (28), this specific result provides some indication that humorous messages are a good strategy to communicate health risk messages to this target audience.

## Limitations

If eye tracker technology is a relevant tool to assess and dissect the effects of humor in a preventive health campaign and adds to direct evidence of the power of humor to attract the participants' attention to the preventive message, our results did not allow us to state whether the enhanced attention devoted to humorous messages reflected (1) the participants' cognitive effort required to understand the humorous content, (2) the affective responses triggered by humor exposure (e.g., experiencing pleasure, fun, happiness), or (3) the fact that participants voluntarily shifted their attention from warnings (i.e., avoid warnings) in conventional nonhumorous health messages (which means less avoidance toward humorous health warning messages). It is a crucial question that offers opportunities for research on visual attention devoted to humorous health messages. Additional research is also needed to examine the relationship between visual attention pattern and behavioral outcomes. If the presence of humor makes it possible to thwart strategies of visual avoidance of warning messages, it could be a first step toward behavior change. Inserting humorous warning messages on tobacco packaging could be a fruitful context to test this idea. Finally, because the current study only examined the effects of humor on two health topics with a female sample, future studies should provide additional evidence on a broader population but also on various health topics. Overall, testing the generalizability of the present finding is a necessary step to ensure that humor is a fruitful message strategy in health communication.

## Data Availability Statement

The raw data supporting the conclusions of this article will be made available by the authors, without undue reservation.

## Ethics Statement

Ethical review and approval was not required for the study on human participants in accordance with the local legislation and institutional requirements. The patients/participants provided their written informed consent to participate in this study.

## Author Contributions

EB and NB contributed to conception and design of the study. AL led eye-tracking data collection. EB and NB performed statistical analysis and wrote the first draft of the manuscript. All authors contributed to the article and approved the submitted version.

## Conflict of Interest

The authors declare that the research was conducted in the absence of any commercial or financial relationships that could be construed as a potential conflict of interest.

## References

[1] Weinberger MG Gulas CS. The emergence of a half-century of research on humour in advertising: What have we learned? Int J Advert. (2019) 38:911–56. 10.1080/02650487.2019.1598831

[2] Hendriks H Strick M. A laughing matter? Health Commun. (2020) 35:1821–9. 10.1080/10410236.2019.1663587 31502474

[3] Lee MJ. The effects of self-efficacy statements in humorous anti-alcohol abuse messages targeting college students: Who is in charge? Health Commun. (2010) 25:638–46. 10.1080/10410236.2010.521908 21153979

[4] Nabi RL. Laughing in the face of fear (of disease detection): Using humor to promote cancer self-examination behavior. Health Commun. (2016) 31:873–83. 10.1080/10410236.2014.1000479 26652312

[5] Ort A Fahr A. The effectiveness of a positively vs. negatively valenced PSA against sexually transmitted diseases: Evidence from an experimental study. Stud Commun Med. (2020) 9:341–66. 10.5771/2192-4007-2020-3-341

[6] Yoon HJ Tinkham SF. Humorous threat persuasion in advertising: The effects of humor, threat intensity, and issue involvement. J Adv. (2013) 42:30–41. 10.1080/00913367.2012.749082

[7] Blanc N Brigaud E. Humor in print health advertisements: Enhanced attention, privileged recognition, and persuasiveness of preventive messages. Health Commun. (2014) 29:669–77. 10.1080/10410236.2013.769832 24160572

[8] Coulson S Kutas M. Getting it: Human event-related brain response to jokes in good and poor comprehenders. Neurosci Lett. (2001) 316:71–4. 10.1016/S0304-3940(01)02387-4 11742718

[9] Goel V Dolan RJ. The functional anatomy of humor: Segregating cognitive and affective components. Nat Neurosci. (2001) 4:237–8. 10.1038/85076 11224538

[10] Suls JM. A two-stage model for the appreciation of jokes and cartoons: an information-processing analyses. In: Goldstein JH McGhee PE editors. The Psychology of Humor: Theoretical Perspectives and Empirical Issues. New York, NY: Academic Press (1972). p. 81–100.

[11] Edwards A Hyde M Masser M. Rapid review of the literature on using humour to reduce negative emotional reactions to unappealing topics. Donor Res Netw. (2020) 1–5. 10.13140/RG.2.2.10290.22721

[12] Sparks JV Lang A. Mechanisms underlying the effects of sexy and humorous content in advertisements. Commun Monog. (2015) 82:134–62. 10.1080/03637751.2014.976236

[13] Skalski P Tamborini R Glazer E Smith S. Effects of humor on presence and recall of persuasive messages. Commun Quart. (2009) 57:136–53. 10.1080/01463370902881619

[14] Futerfas ML Nan X. Role of humor in the persuasiveness of entertainment narratives on unprotected sexual behavior. J Health Commun. (2017) 22:312–8. 10.1080/10810730.2017.1284285 28276946

[15] Moyer-Gusé E Mahood C Brookes S. Entertainment-education in the context of humor: Effects on safer-sex intentions and risk perceptions. Health Commun. (2011) 26:765–74. 10.1080/10410236.2011.566832 21707390

[16] Young DG. The privileged role of the late-night joke: Exploring humor's role in disrupting argument scrutiny. Med Psychol. (2008) 11:119–42. 10.1080/15213260701837073

[17] Moyer-Gusé E Robinson M McKnight J. The role of humor in messaging about the MMR vaccine. J Health Commun. (2018) 23:514–22. 10.1080/10810730.2018.1473533 29757123

[18] Casado-Aranda L Sánchez-Fernández J Ibáñez-Zapata J. Evaluating communication effectiveness through eye tracking: benefits, state of the art, and unresolved questions. Int J Bus Commun. (2020) 1–38. 10.1177/2329488419893746

[19] Meernik C Jarman K Wright ST Klein EG Godstein AO Ranney L. Eye tracking outcomes in tobacco control regulation and communication: A systematic review. Tob Reg Sci. (2016) 2:377–403. 10.18001/TRS.2.4.9 27668270 PMC5033068

[20] Chou W Trivedi N Peterson E Gaysynsky A Krakow M Vraga E. How do social media users process cancer prevention messages on facejournal? An eye-tracking study. Pat Educ Couns. 103:1161–67. 10.1016/j.pec.2020.01.013 32044193

[21] Meppelink CS Bol N. Exploring the role of health literacy on attention to and recall of text-illustrated health information: an eye-tracking study. Comp Hum Behav. (2015) 48:87–93. 10.1016/j.chb.2015.01.027

[22] Yzer M Han J Choi K. Eye movement patterns in response to anti-binge drinking messages. Health Commun. (2018) 33:1454–61. 10.1080/10410236.2017.1359032 28850248 PMC5832498

[23] Strick M Holland RW van Baaren R van Knippenberg A. Humor in the eye tracker: Attention capture and distraction from context cues. J Gen Psychol. (2010) 137:37–48. 10.1080/00221300903293055 20198815

[24] Carter BT Luke SG. Best practices in eye tracking research. Int J Psychophysiol. (2020) 155:49–62. 10.1016/j.ijpsycho.2020.05.010 32504653

[25] Holmqvist K Nyström M Andersson R Dewhurst R Jarodzka H Van de Weijer J. Eye Tracking: A Comprehensive Guide to Methods and Measures. Oxford: Oxford University Press. (2011).

[26] Joseph A Murugesh R. Potential eye tracking metrics and indicators to measure cognitive load in human-computer interaction research. J Sci Res. (2020) 64:168–75. 10.37398/JSR.2020.640137

[27] Adil S Lacoste-Badie S Droulers O. Face presence and gaze direction in print advertisements. How they influence consumer responses: An eye-tracking study. J Adv Res. (2018) 58:443–5. 10.2501/JAR-2018-004

[28] Olié V Pasquereau A Assogba F Arwidson P Nguyen-Thanh V Chatignoux E. Changes in tobacco-related morbidity and mortality in French women: worrying trends. Eur J Public Health. (2020) 30:380–5. 10.1093/eurpub/ckz171 31711145

[29] Maynard OM Attwood A O'Brien L Brooks S Hedge C Leonards U. Avoidance of cigarette pack health warnings among regular cigarette smokers. Drug Alcohol Dep. (2014) 136:170–4. 10.1016/j.drugalcdep.2014.01.001 24485554 PMC4959561

[30] Maynard OM Munafò MR Leonards U. Visual attention to health warnings on plain tobacco packaging in adolescent smokers and non-smokers. Addiction. (2013) 108:413–9. 10.1111/j.1360-0443.2012.04028.x 22882736

[31] Sillero-Rejon C Leonards U Munafò MR Hedge C Hoek J Toll B. Avoidance of tobacco health warnings? An eye-tracking approach. Addiction. (2021) 116:126–38. 10.1111/add.15148 32506597

